# Efficacy of Bovine Respiratory Syncytial Virus Vaccines to Reduce Morbidity and Mortality in Calves Within Experimental Infection Models: A Systematic Review and Meta-Analysis

**DOI:** 10.3389/fvets.2022.906636

**Published:** 2022-06-15

**Authors:** David A. Martinez, Benjamin Newcomer, Thomas Passler, Manuel F. Chamorro

**Affiliations:** ^1^Department of Clinical Sciences, College of Veterinary Medicine, Auburn University, Auburn, AL, United States; ^2^Department of Large Animal Clinical Sciences, College of Veterinary Medicine and Biomedical Sciences, West Texas A&M University, Canyon, TX, United States

**Keywords:** BRSV challenge, respiratory disease, vaccination efficacy, calves, morbidity and mortality

## Abstract

Producers and veterinarians commonly use vaccination as the main strategy to reduce the incidence of bovine respiratory syncytial virus (BRSV) infection in calves; however, supportive evidence of BRSV vaccination efficacy has been inconsistent in the literature. The objective of this meta-analysis was to evaluate data from controlled studies on the efficacy of commercially available BRSV vaccines on reducing calf morbidity and mortality after experimental infection with BRSV. A systematic review and meta-analysis was performed in BRSV experimental challenge studies that reported the efficacy of commercially available modified-live virus (MLV) and inactivated BRSV vaccines on protection against calf morbidity and mortality. The studies included in the analysis were randomized, controlled, clinical trials with clear definitions of calf morbidity and mortality. Risk ratios with 95% confidence intervals and forest plots were generated. Fourteen studies including 29 trials were selected for the analysis. Commercially available MLV BRSV vaccines reduced the risk of calf mortality after experimental infection with BRSV. Modified-live virus vaccines reduced the risk of morbidity in calves with absence of serum maternal antibodies at initial vaccination, but failed to demonstrate significant morbidity reduction when calves were vaccinated in the face of maternal immunity. Results from experimental challenge studies do not always represent the conditions of natural infection and caution should be used when making vaccine recommendations.

## Introduction

Bovine respiratory syncytial virus (BRSV) is considered one of the most important viral pathogens of the bovine respiratory disease complex (BRDC) in calves (1). A high seroprevalence of BRSV in the United States (US) ranging from 60 to 80% has been reported (2), and BRSV-associated respiratory disease outbreaks are described in dairy and beef calves (1–3). Bovine respiratory syncytial virus most commonly affects calves under 6 months of age with moderate to high morbidity rates and low mortality rates; however, the case fatality rate can be as high as 31% in some cases (4, 5). The severity of disease following BRSV infection depends on host immunity. Clinical signs of infection, as well as overall morbidity and mortality rates, are lower when calves have moderate to high levels of BRSV neutralizing serum antibodies, regardless if those were acquired passively through maternal colostrum or actively generated by prior BRSV infection or vaccination (6–8). Different experimental challenge and natural infection studies have been conducted to determine the efficacy of BRSV vaccination protocols to prevent BRSV infection and disease in calves (4, 5, 9–13). Results from a previous meta-analysis suggested a lack of efficacy of BRSV vaccination on the reduction of morbidity and mortality associated with bovine respiratory disease (9). However, during the past decade, several randomized clinical trials evaluating clinical protection afforded by calf vaccination demonstrated moderate protection after experimental infection with BRSV (7, 10, 11, 14, 15). Results from these studies suggest that a significant reduction in the titers and duration of BRSV shedding after challenge is observed in vaccinated calves compared with non-vaccinates; however, significant and consistent reduction of respiratory clinical scores in calves is variable among published BRSV vaccination efficacy studies (12, 14). The first objective of this study was to perform a systematic review and meta-analysis of published literature to evaluate if dairy and beef calves under 6 months of age vaccinated with commercially available BRSV vaccines had lower risk ratios of becoming sick or dying after experimental challenge with BRSV, compared with non-vaccinated control calves. The second objective of this study was to determine if the presence of BRSV serum neutralizing antibodies at the time of initial vaccination was associated with morbidity and mortality outcomes. The overarching goal of this meta-analysis was to provide BRSV calf-vaccination efficacy information to the veterinary community.

## Materials and Methods

This systematic review and meta-analysis was performed following PRISMA 2020 guideline recommendations. Studies in the English language that reported the efficacy of commercially available BRSV vaccines in calves undergoing experimental challenge with BRSV published in peer reviewed scientific journals were identified. The literature search, the inclusion/exclusion criteria, and data extraction was performed by all authors following an objective protocol agreed upon prior the start of the meta-analysis. The literature search was performed in March 2021 using four scientific databases with no publication date restrictions, namely PubMed, CAB, Web of Science, and Agricola using the keywords “viral” or “virus”, “bovine” or “calf” or “cattle”, “vaccine” or “immunization”, and “respiratory” or “BRD”, or “BRSV”. After the initial search, review articles, book chapters, and duplicated articles were excluded from the analysis. When available, filters were used to exclude review articles during the search. Articles collected from the database search were included in the meta-analysis based on the following criteria: 1. The article was relevant to the study objectives; 2. The article was a randomized clinical trial and included a non-vaccinated/sham vaccinated control group; 3. The article described the use of a commercially-available BRSV-containing vaccine; 4. The article involved experimental infection with BRSV; 5. The article reported clinically relevant and well-defined outcomes (i.e., morbidity and mortality rates). Articles were excluded from the analysis if the four authors agreed they did not meet the inclusion criteria.

Relevant data for outcome analysis such as the total number of sick calves (morbidity) and the total number of dead calves (mortality) after experimental infection with BRSV was extracted from all articles and their respective trials. Only studies using commercially available BRSV-containing vaccines were used in this meta-analysis because of their relevance on providing practicing veterinarians information applicable to their clients. To evaluate the morbidity risk, the number of calves with signs of respiratory disease associated with BRSV challenge such as abnormal respiratory rate and effort, abnormal rectal temperature, nasal discharge, abnormal mentation (lethargy), the presence of cough, and the presence of abnormal lung sounds (crackles and wheezes) were noted for each trial. To evaluate mortality risk, the number of calves reported as dead following experimental infection with BRSV or humanely euthanized due to the severity of respiratory disease because of experimental infection with BRSV were noted. If calves were euthanized during any portion of the study for reasons different from a humane endpoint associated with BRSV infection following challenge, mortality data were not included in the analysis. Data were analyzed using a commercially available meta-analysis software (CMA, Biostat, Englewood, NJ, USA). To compare the probability of an outcome in exposed (vaccinated) groups with the probability of the same outcome occurring in the non-exposed (non-vaccinated) groups, the risk ratio (RR) and 95% CI for each outcome were used as effect size. The heterogeneity among studies or trials was assessed by the Cochran Q statistic, with *P* ≤ 0.10, and *I*^2^ statistic > 50% indicating heterogeneity. A random effects model was used to compare mean effect size across treatment groups and forest plots were constructed for each meta-analysis. The forest plots generated by the software excluded trials without a significant effect on each outcome (i.e., equal morbidity and/or mortality rates in animals from exposed and non-exposed groups). Summary measures were considered significantly different between treatment groups if the 95% CI did not include 1. In some studies, the same control group was used in different trials. Publication bias was visually assessed using funnel plots of the standard error by log risk ratio.

The type of vaccine used and the presence of BRSV serum neutralizing antibodies of maternal origin at the time of initial vaccination were assessed in all studies. For studies evaluating MLV vaccines, the effect of route of administration was evaluated. To determine the effect of vaccination, the effect of type of vaccine (MLV vs. inactivated), effect of the presence or absence of BRSV antibodies at the time of initial vaccination, and effect of route of MLV vaccine administration (intranasal vs. parenteral), quantitative syntheses were performed within each outcome using a subset of studies relevant to that outcome. The final meta-analysis included separate evaluations of morbidity and mortality outcomes from vaccination in general, vaccination with MLV vaccines, and vaccination with inactivated vaccines. The effect of maternal antibodies on morbidity and mortality outcomes was evaluated for each separate analysis.

## Results

**Figure 1:**
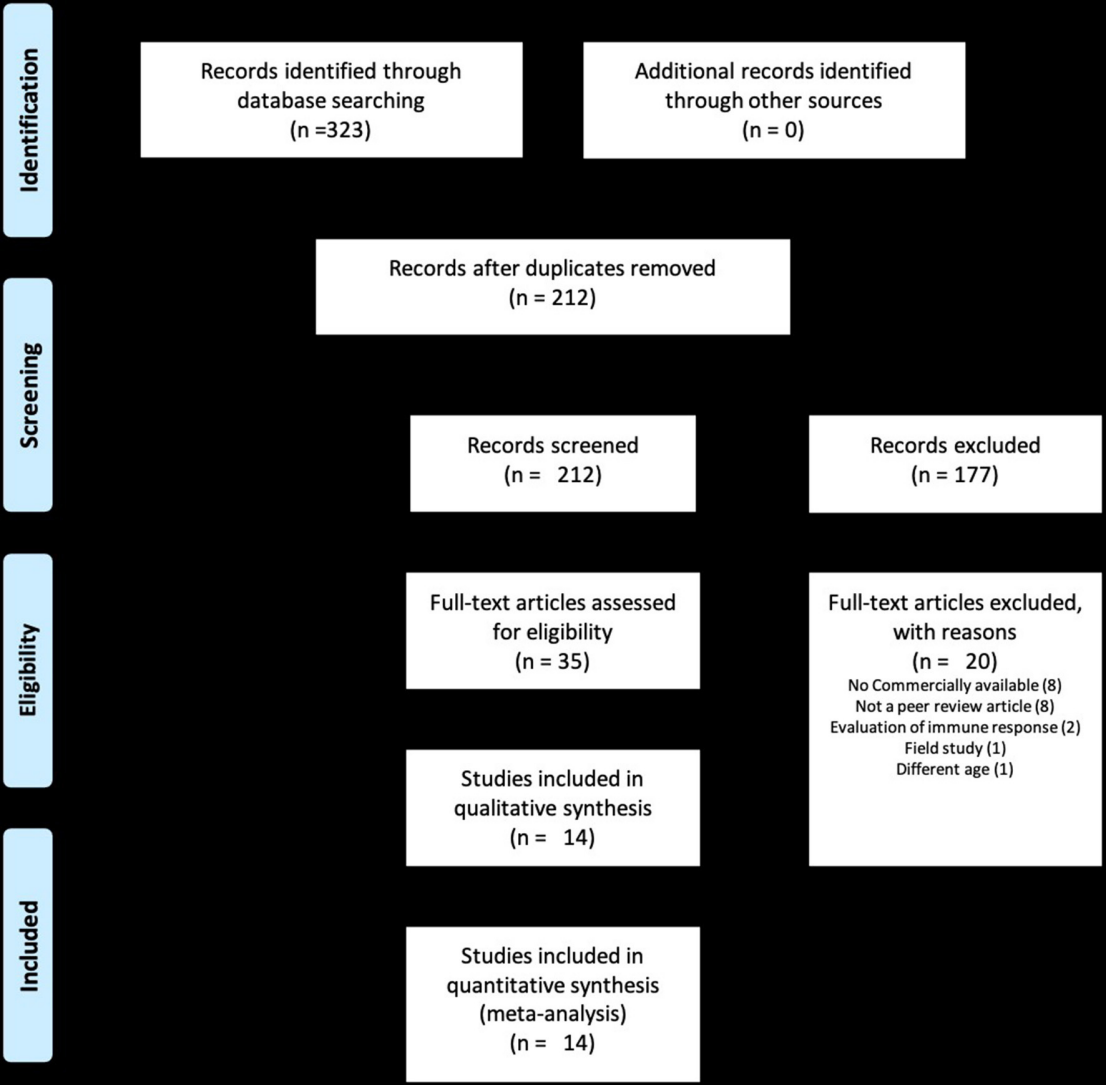
PRISMA flow diagram of studies included in the analysis.

The total number of studies identified in the initial literature search was 323. Following evaluation of abstracts and complete review of articles eligible for the study, 14 studies comprising 29 different trials were selected for the meta-analysis (Figure 1) (4, 7–9, 12–21). Studies excluded from the analysis included 309 articles. The reasons for exclusion were that studies were not a randomized clinical trial, were not a peer-reviewed article, did not include commercially available vaccines, or did not include clinically relevant outcomes of morbidity and mortality such as number of animals with signs of respiratory disease after experimental challenge, number of animals dying after experimental challenge, and clinical respiratory scores. Within the 29 trials from the 14 studies, five evaluated inactivated vaccines, 23 evaluated MLV vaccines, and 1 evaluated an MLV vaccine followed by a booster with an inactivated vaccine (Table 1). While calves in some of the trials received only a single vaccine dose, calves in other studies were also administered a booster. Visual assessment of the funnel plots demonstrated an approximately symmetric inverted funnel shape distribution of the data points which is the pattern expected when publication bias is unlikely (data not shown).

**Table 1:** Studies evaluating the effect of vaccination on clinical protection against experimental infection with BRSV in calves.

**References**	**Vacc. type**	**Route**	**Study calves**	**Maternal antibodies**	**Age at vaccination (days)**	**Booster (days)**	**Challenge after vacc. (days)**	**Follow up time (days)**
Xue et al. (22)	MLV	IN	Dairy	No	5.5	NA	21	14
West et al. (17)-1	MLV	SC	Dairy	No	21	21	21	8
West et al. (17)-2	MLV	SC	Dairy	No	21	NA	21	8
West et al. (17)-3	MLV	SC	Dairy	No	21	NA	21	8
Ellis et al. (23)-1	MLV	IN	Dairy	No	42	21	21	8
Ellis et al. (23)-2	MLV	IN	Dairy	No	63	NA	21	8
Ellis et al. (23)-3	MLV	IN	Dairy	No	14	NA	8	8
Vangeel et al. (24)-1	MLV	IN	Dairy	No	21	NA	21	8
Vangeel et al. (24)-2	MLV	IN	Dairy	No	21	NA	10	8
Vangeel et al. (24)-3	MLV	IN	Dairy	No	21	NA	5	8
Vangeel et al. (24)-4	MLV	IN	Dairy	Yes	21	NA	66	8
Ellis et al. (11)-1	MLV	IN	Dairy	Yes	5.5	NA	135	8
Ellis et al. (11)-2	MLV	IN	Dairy	No	5.5	NA	135	8
Ellis et al. (11)-3	MLV	IN	Dairy	No	5.5	NA	21	8
Ellis et al. (11)-4	MLV	SC	Dairy	No	5.5	NA	21	8
Ellis et al. (7)-1	MLV	IN	Dairy	No	6	NA	49	8
Ellis et al. (7)-2	MLV	IN	Dairy	Yes	7	NA	63	8
Ellis et al. (7)-3	MLV	IN	Dairy	Yes	7	NA	105	8
Ellis et al. (7)-4	MLV	IN	Dairy	Yes	9	NA	77	8
Gray et al. (15)-1	MLV	IN	Dairy	No	7	NA	42	8
Gray et al. (15)-2	MLV	IN	Dairy	No	7	NA	42	8
Kolb et al. (12)	MLV	SC	Dairy	Yes	30	NA	90	8
Ellis et al. (18)-1	KV	SC	Dairy	Yes	63	21	42	8
Patel and Didlick (25)-1	KV	SC	Mix	No	14	21	126	14
Patel and Didlick (25)-2	KV	SC	Mix	No	14	21	266	14
Ellis et al. (26)	KV	SC	Dairy	No	63	20	46	8
Ellis et al. (14)-1	MLV/MLV	IN/SC	Beef	Yes	1	60	120	7
Ellis et al. (14)-2	MLV/KV	IN/SC	Beef	Yes	1	60	120	7
van der Sluijs et al. (13)	KV	SC	Dairy	Yes	14	NA	28	35

*The numbers 1, 2, 3, 4 provided in front of some references are correspond to the trial number in each study*.

### BRSV-Vaccination

**Figure 2:**
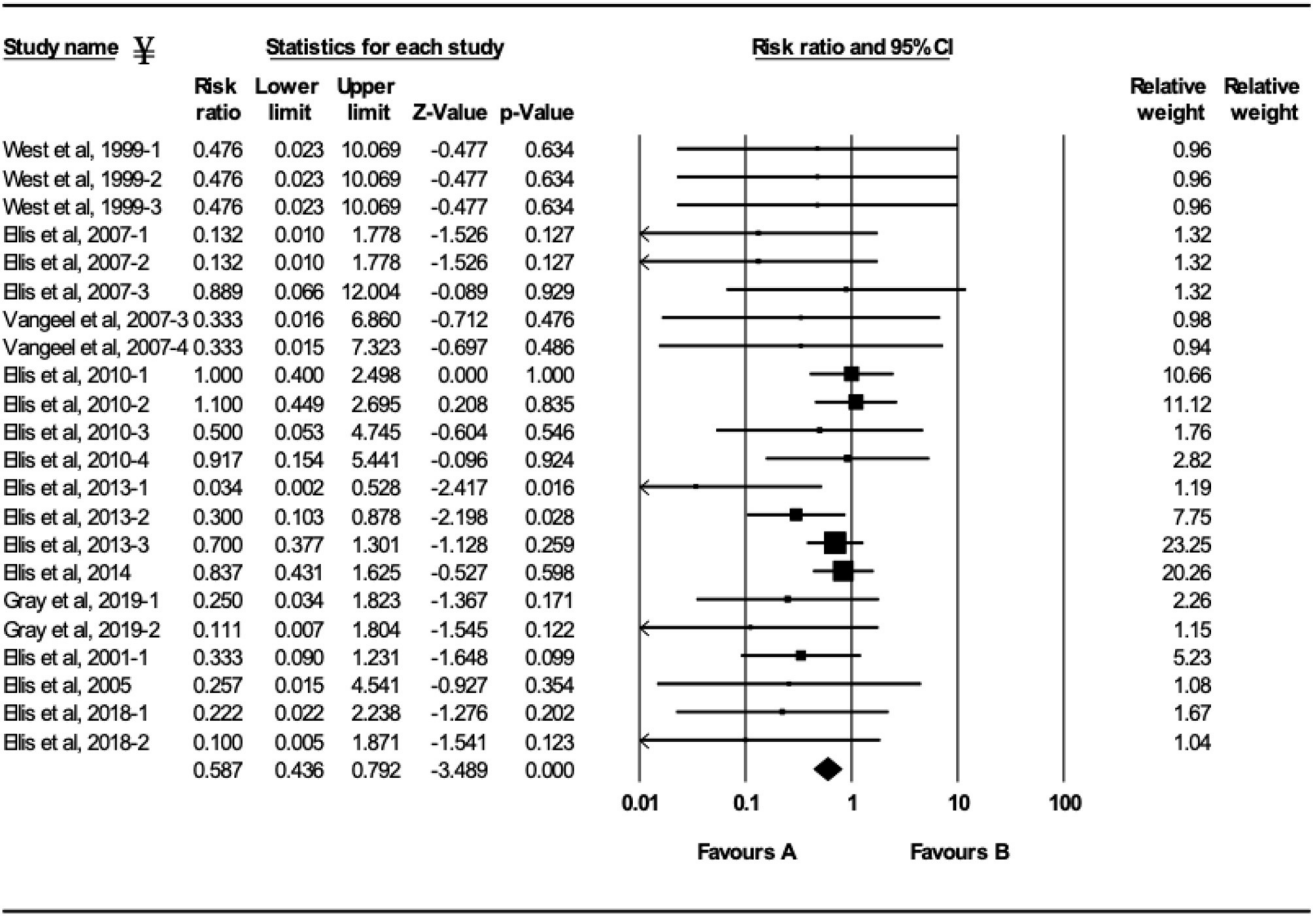
Forest plot of mortality risk ratios from experimental BRSV challenge trials that evaluated MLV and inactivated BRSV vaccines. ^¥^Only trials with a significant mortality effect between vaccinated and control calves are shown by the meta-analysis software in the forest plot. Heterogeneity stats: *Q*-Value: 18.64, df (*Q*): 21 *p*: 0.61 *I*^2^: 0.

**Figure 3:**
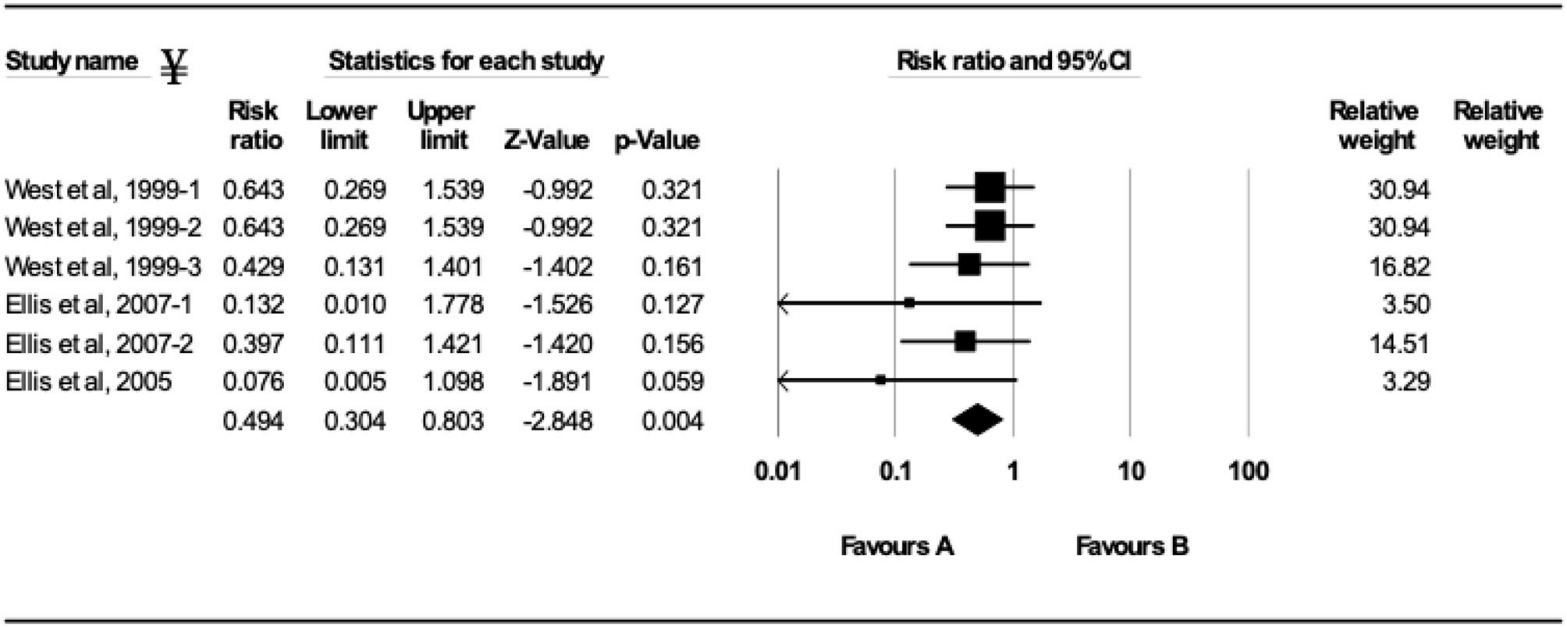
Forest plot of morbidity risk ratios from experimental BRSV challenge trials that evaluated MLV and inactivated BRSV vaccines in seronegative calves (absence of maternal antibodies at initial vaccination). ^¥^Only trials with a significant morbidity effect between vaccinated and controls calves are shown by the meta-analysis software in the forest plot. Heterogeneity stats: *Q*-Value: 6.19, df (*Q*): 6 *p*: 0.40 *I*^2^: 3.21.

**Figure 4:**
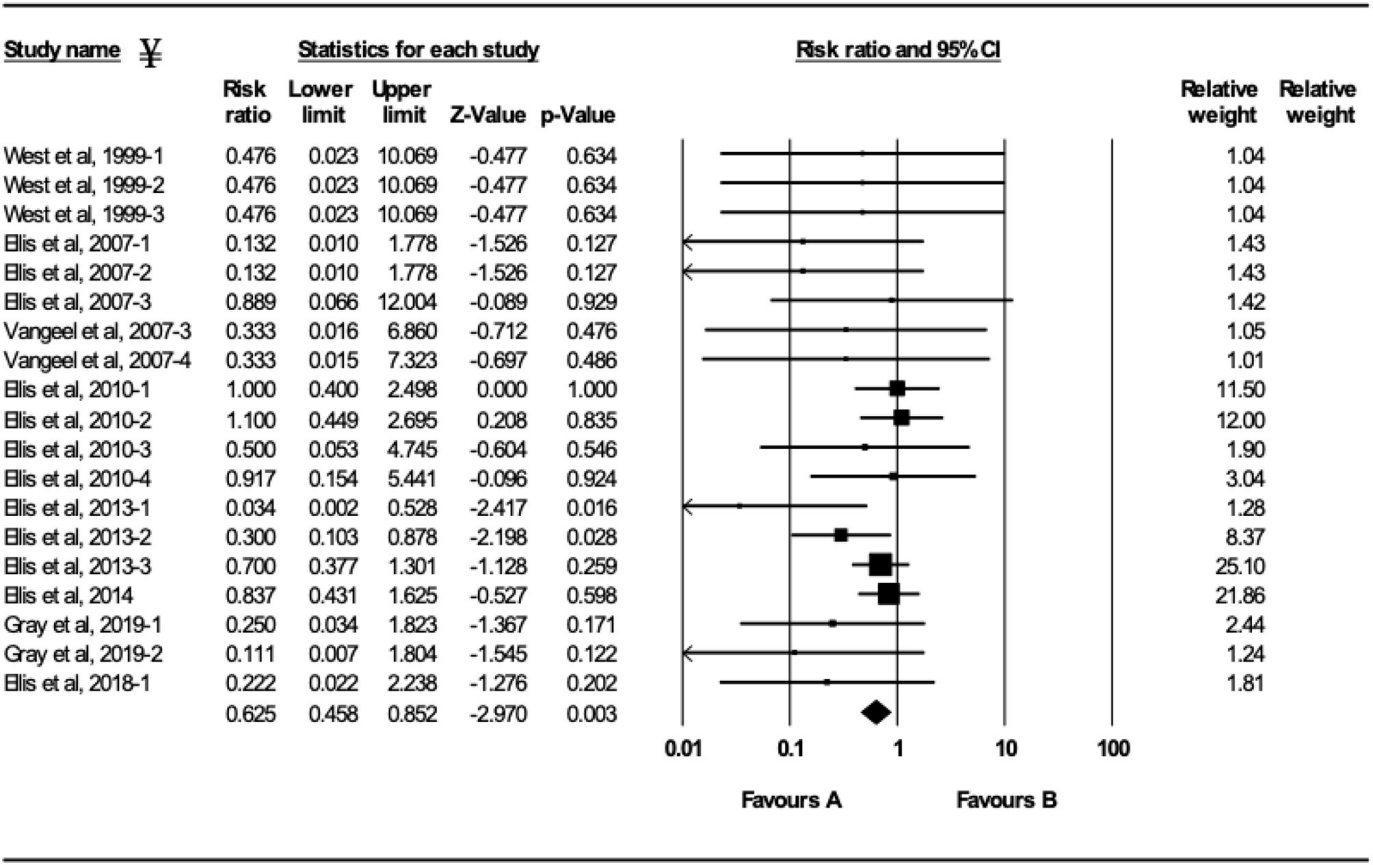
Forest plot of mortality risk ratios from experimental BRSV challenge trials that evaluated MLV BRSV vaccines. ^¥^Only trials with a significant mortality effect between vaccinated and controls calves are shown by the meta-analysis software in the forest plot. Heterogeneity stats: *Q*-Value: 16.04, df (*Q*): 18 *p*: 0.59 *I*^2^: 0.

**Figure 5:**
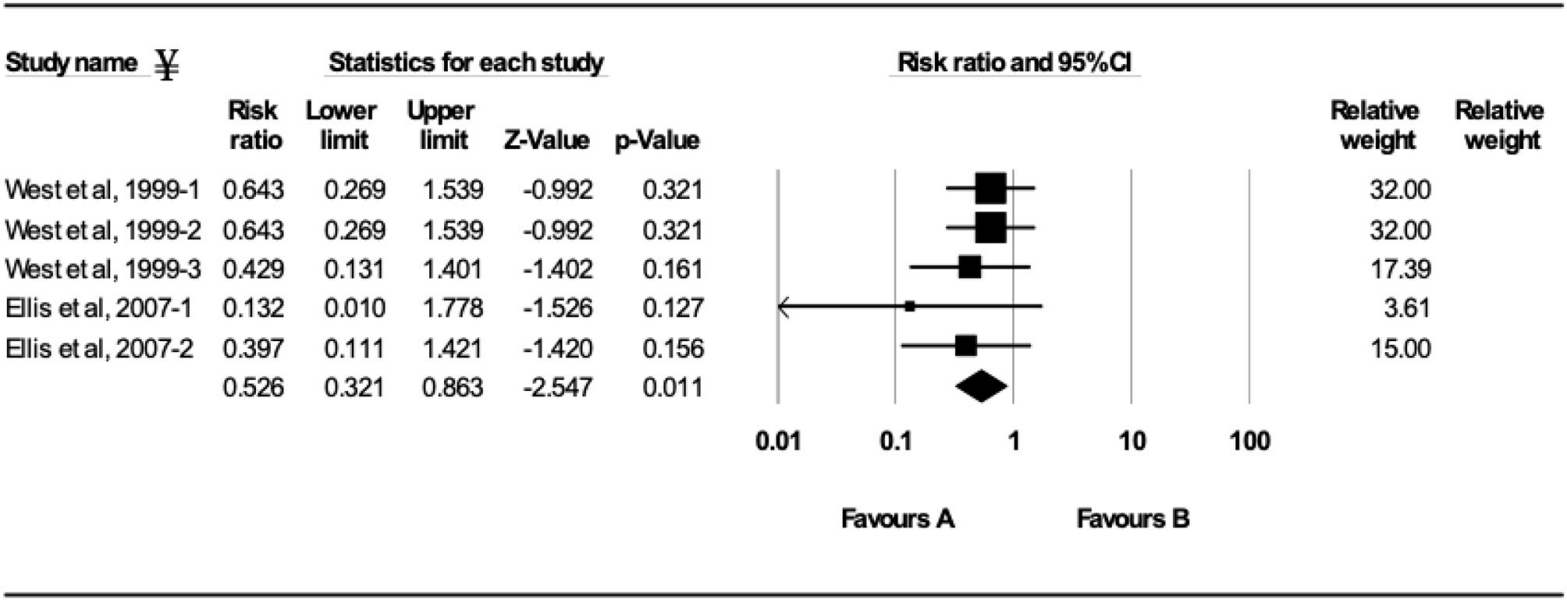
Forest plot of morbidity risk ratios from experimental BRSV challenge trials that evaluated MLV vaccines in seronegative calves (absence of maternal antibodies at initial vaccination). ^¥^Only trials with a significant morbidity effect between vaccinated and controls calves are shown by the meta-analysis software in the forest plot. Heterogeneity stats: *Q*-Value: 3.55, df (*Q*): 5, *p*: 0.62, *I*^2^: 0.

The efficacy of vaccination either with MLV or inactivated BRSV vaccines on clinical protection of beef and dairy calves against experimental BRSV challenge was evaluated in 29 trials from 14 different studies (Figures 2–5). Of the 29 trials, 10 reported the presence of maternal antibodies at initial vaccination of study calves and 19 reported absence of maternal antibodies at initial vaccination of study calves (4, 7–9, 12–21). The analysis demonstrated a 41.3% reduction in the mortality risk (RR = 0.587; 95% CI 0.436–0.792) for vaccinates, compared with controls independently of type of vaccine or the presence or absence of maternal antibodies at initial vaccination (Figure 2). A 50.6% reduction of the morbidity risk (RR = 0.494; 95% CI 0.304–0.803) was demonstrated in trials in which calves had no serum neutralizing antibodies of maternal origin at initial vaccination (Figure 3). In trials in which calves had serum neutralizing antibodies at initial vaccination, the morbidity risk was not significantly different between vaccinates and controls.

### Vaccination With MLV BRSV Vaccines

Ten studies comprising 23 trials evaluated the effect of MLV vaccination on clinical protection of beef and dairy calves against experimental infection with BRSV (4, 7, 9, 12, 16–21). Within the 23 trials, calves from 7 trials had serum maternal antibodies at initial vaccination. In contrast, calves from 16 trials did not have serum maternal antibodies at initial vaccination. A 37.5% reduction of the mortality risk (RR = 0.625; 95% CI 0.458–0.852) for vaccinates compared with controls was demonstrated independently of the presence or absence of maternal antibodies at initial vaccination and route of administration (Figure 4). A 47.4% reduction of the morbidity risk (RR = 0.526; 95% CI 0.321–0.863) in vaccinates compared with controls was demonstrated in trials in which calves had no titers of maternal antibodies at initial vaccination independently of route of administration (Figure 5). In trials in which calves had maternal antibodies at initial vaccination, the analysis did not demonstrate a significant reduction of the morbidity risk in vaccinates compared with controls regardless route of vaccine administration (intranasal = 4 trials vs. parenteral = 2 trials).

### Vaccination With Inactivated-BRSV Vaccines

Four studies including five different trials evaluated clinical protection afforded by vaccination of calves with inactivated vaccines against experimental infection with BRSV (8, 13–15). Similar morbidity and mortality risks in vaccinates compared with controls were demonstrated by the analysis. The number of trials using inactivated BRSV vaccines and reporting the presence or absence of maternally derived immunity at initial vaccination was insufficient to evaluate the effect of maternal antibodies on morbidity and mortality outcomes following vaccination and challenge.

## Discussion

This meta-analysis provides quantitative information about the efficacy of commercially available BRSV vaccines reducing calf morbidity and mortality following experimental BRSV infection. In this meta-analysis, vaccination of calves under 6 months of age with MLV BRSV vaccines demonstrated a significant reduction of the mortality risk associated with experimental BRSV infection; however, reduction of the morbidity risk was demonstrated only for calves that did not have serum neutralizing antibodies at the time of vaccination. Vaccination of calves under 6 months of age with inactivated BRSV vaccines did not result in significant reduction of morbidity or mortality risks associated with experimental BRSV infection. Our results contrast with the results of a previous meta-analysis (9). Theurer et al. (9) reported that no significant reduction of the morbidity and mortality risks after experimental infection with BRSV in calves previously vaccinated with MLV BRSV vaccines. The authors of that study commented that a greater study heterogeneity in studies involving MLV vaccines could have limited their ability to detect a significant effect on morbidity and mortality among MLV-vaccinated and control calves. Interestingly, results from that same study (9) demonstrated a significant reduction of calf mortality after experimental BRSV challenge in calves vaccinated with an inactivated BRSV vaccine. It is possible that lack of power and greater chance for making a type II error resulting from a low number of trials using inactivated vaccines in this meta-analysis prevented the observation of significant differences on calf morbidity and mortality in studies involving inactivated vaccines. Additionally, results from previous studies suggest that incomplete and short-lived humoral and cell-mediated immune memory responses induced by inactivated vaccines could be associated with their lack of significant effects on calf morbidity and mortality in BRSV experimental challenge trials (16, 17). Although BRSV is a single piece of the puzzle in the natural occurrence of the bovine respiratory disease complex (BRDC), its seroprevalence in US cattle populations is high, and case-fatality rates of acutely infected cattle can reach 31% in some cases (5). Therefore, the efficacy of MLV BRSV vaccines on reducing calf mortality following experimental BRSV infection provides evidence of the importance of the BRSV component in vaccination programs aimed to control the natural occurrence of BRDC in cattle.

In studies where the effect of vaccination was evaluated in calves devoid of BRSV-specific serum maternal antibodies, the lack of maternal immunity was associated with reduction of calf morbidity after experimental BRSV infection in vaccinates compared with controls. It is possible that the absence of maternal interference favored the induction of complete immune responses to vaccination and prevented clinical disease in vaccinates. In contrast, in studies where the effect of vaccination was evaluated in calves with BRSV-specific serum maternal antibodies, the presence of maternal immunity was not associated with significant reduction of calf morbidity in vaccinates. It is possible that the presence of serum maternal antibodies similarly prevented clinical disease after experimental BRSV infection in vaccinated and control calves in these studies. Results from previous studies have demonstrated that the presence of maternal immunity suppress local and systemic antibody responses following vaccination and provide clinical protection against experimental infection with respiratory viruses in calves (6, 8, 11, 18–21).

The most important limitation of this meta-analysis is that it was restricted to evaluation of vaccination efficacy in experimental BRSV challenge studies. The extrapolation of vaccine efficacy data from experimental challenge studies to field conditions requires caution; however, randomized, controlled vaccination efficacy studies under natural disease occurrence conditions do not exist in the literature, specifically with respect to BRSV. Several reasons, including the need of a considerable sample size of the target population, the need of a control/unvaccinated group (unlikely in client-owned cattle where the numbers might be adequate), the poly-microbial nature in the etiology of the BRDC, and funding limitations could explain the scarcity of these type of studies. Although vaccination efficacy analysis from experimental challenge studies might not be ideal, it provides meaningful information for practicing veterinarians in the decision-making and vaccine recommendation process. The BRDC continues to be the most economically important disease affecting cattle operations in the U.S., and BRSV is as an important etiologic agent of the complex. Therefore, experimental challenge BRSV vaccination-efficacy studies might be the only alternative for veterinarians to make evidence-based vaccination recommendations for the prevention of BRSV infection in cattle. Other limitations included the limited number of studies evaluating inactivated-BRSV vaccines and the use of the same control group in different trials (from the same study) within the same study. It is possible that some experimental challenge studies using inactivated BRSV vaccines were excluded from this analysis because of failure to meet the selection criteria. In some of these studies, exacerbation of BRSV infection and clinical disease associated with a Th2-driven immune response (BRSV-specific IgE and histamine production) was reported in calves previously vaccinated with inactivated BRSV vaccines (4, 5, 21). Other inactivated vaccine studies evaluating recombinant protein/subunit vaccines were not included in the analysis because these types of vaccines are not commercially available. In order to include a greater number of trials for this meta-analysis the control group for each study was re-used within the analysis of data.

## Conclusion

Results from this meta-analysis suggest that MLV BRSV vaccination reduces calf mortality following experimental BRSV infection. Additionally, vaccination of seronegative calves with MLV BRSV vaccines reduced calf morbidity and mortality following experimental BRSV infection. Based on this study's findings, we conclude that vaccination of calves with failure in the transfer of passive immunity, colostrum-deprived, or with lack of passive BRSV antibodies is important for prevention of clinical disease associated with BRSV infection; however, veterinarians need to use caution when recommending vaccination protocols against BRDC based on results from experimental viral infection studies.

## Data Availability Statement

The original contributions presented in the study are included in the article/supplementary material, further inquiries can be directed to the corresponding author/s.

## Author Contributions

DM, BN, TP, and MC contributed equally with the study design, developing of the systematic review protocol, literature search strategy and implementation, selection of articles, data evaluation and extraction, and data analysis for this study. DM took the lead on writing the manuscript product of this study. BN, TP, and MC participated on manuscript review and edition before submission. All authors contributed to the article and approved the submitted version.

## Funding

Funding for publication fees will be associated with MC's start-up funds at Auburn University.

## Conflict of Interest

The authors declare that the research was conducted in the absence of any commercial or financial relationships that could be construed as a potential conflict of interest.

## Publisher's Note

All claims expressed in this article are solely those of the authors and do not necessarily represent those of their affiliated organizations, or those of the publisher, the editors and the reviewers. Any product that may be evaluated in this article, or claim that may be made by its manufacturer, is not guaranteed or endorsed by the publisher.
